# Development of an effective risk management system in a teaching hospital

**DOI:** 10.1186/2251-6581-11-15

**Published:** 2012-09-21

**Authors:** Hossein Adibi, Nader Khalesi, Hamid Ravaghi, Mahdi Jafari, Ali Reza Jeddian

**Affiliations:** 1 Department of Health Services Management, School of Health Management and Information Sciences, Tehran University of Medical Sciences, Tehran, Iran; 2 Shariaty Hospital, Tehran University of Medical Sciences, Tehran, Iran

**Keywords:** Patient safety, Risk management, Adverse event

## Abstract

**Background:**

Unsafe health care provision is a main cause of increased mortality rate amongst hospitalized patients all over the world. A system approach to medical error and its reduction is crucial that is defined by clinical and administrative activities undertaken to identify, evaluate, and reduce the risk of injury. The aim of this study was to develop and implement a risk management system in a large teaching hospital in Iran, especially of the basis of WHO guidelines and patient safety context.

**Methods:**

WHO draft guideline and patient safety reports from different countries were reviewed for defining acceptable framework of risk management system. Also current situation of mentioned hospital in safety matter and dimensions of patient safety culture was evaluated using HSOPSC questionnaire of AHRQ. With adjustment of guidelines and hospital status, the conceptual framework was developed and next it was validated in expert panel. The members of expert panel were selected according to their role and functions and also their experiences in risk management and patient safety issues. The validated framework consisted of designating a leader and coordinator core, defining communications, and preparing the infrastructure for patient safety education and culture-building. That was developed on the basis of some values and commitments and included reactive and proactive approaches.

**Results:**

The findings of reporting activities demonstrated that at least 3.6 percent of hospitalized patients have experienced adverse events and 5.3 percent of all deaths in the hospital related with patient safety problems. Beside the average score of 12 dimensions of patient safety culture was 46.2 percent that was considerably low. The “non-punitive responses to error” had lowest positive score with 21.2 percent.

**Conclusion:**

It is of paramount importance for all health organizations to lay necessary foundations in order to identify safety risks and improve the quality of care. Inadequate participation of staff in education, reporting and analyzing, underreporting and uselessness of aggregated data, limitation of human and financial resources, punitive directions and management challenges for solutions were the main executive problems which could affect the effectiveness of system.

## Background

Unsafe and potentially life threatening health care provision is a main cause of death and increased mortality rate amongst hospitalized patients in different countries [1,2]. Results of different studies have shown that a substantial number of patients are affected or even die as a result of defective health care in hospitals [3-18]. Adverse events in hospitals are now widely agreed to be a serious problem, annually killing more people than breast cancer or AIDS [19]. Studies revealed that 2.9% to 16.6% of patients suffer from at least one of such complications and 5% to 13% of them die as a result. It is estimated that some 50% of this complications can be prevented [3,15,17]. Measuring the indicators and extent of adverse events may create a sense of urgency for Systematic intervention [1,2]. It is generally believed that errors and mismanagements of patients are directly related to defects and insufficiencies of the health care system and in many cases, they are originated from similar defects in the system [18].

A system approach to medical error and its reduction is crucial. To meet such important objective, establishment of a risk management system is necessary [20]. Risk management in health care is defined by clinical and administrative activities undertaken to identify, evaluate, and reduce the risk of injury to patients, staff, and visitors and the risk of loss to the organization itself [21]. 7 steps in the Risk Management process are establishment the context, identifying, analyzing, evaluating, and treating the risks, continuous monitoring and review, and communication and consultation [22].

This study aimed to develop and implement a risk management system in a large teaching hospital, specifically according to World Health Organization guidelines and patient safety reports. As to our knowledge, it was the first such experience of its type in Iran and we aimed to assess the limitations and insufficiencies of it. Analysis of results and findings of implementation of this system will be presented in separate articles.

## Materials and methods

### Conceptual framework

WHO draft guideline that is published in world alliance for patient safety program and patient safety reports from different countries were reviewed for defining acceptable framework of risk management system. Also current situation of mentioned hospital which is a large teaching hospital with more than 600 beds and expert university faculties, was evaluated in safety matter by direct interview with medical, nursing and management staff, and with focus group discussion in clinical governance committee and visit of wards and divisions. With adjustment of guidelines and hospital status, the conceptual framework was developed and next, it was validated in expert panel including senior hospital management and deputy managers of health care, education, supportive affaires; operation rooms and emergency ward managers; clinical governance committee; nursing supervisors; and faculty staff that involved in mortality and morbidity boards and other safety issues. The members of expert panel were selected according to their role and functions and also their experiences in risk management and patient safety areas.

For validation of the model and gathering of expert opinions, a likert questionnaire was used whose content validity approved by experts and its reliability was assessed by Cronbach’s Alpha coefficient. The Cronbach's Alpha coefficient equal to 0.76 shows appropriate internal consistency and reliability of the questionnaire for validation of the model.

**Figure 1:**
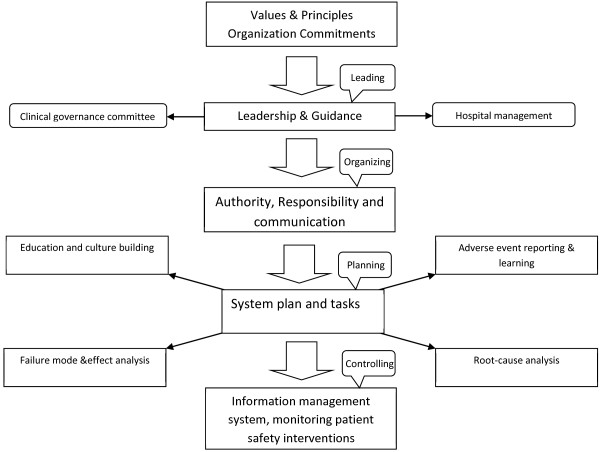
The Validated model for risk management system.

The framework is shown in Figure 1. The policy development and executive program in risk management system consisted of designating a leader and coordinator core and defining its role, and defining communications with hospital boards and committees, describing processes and preparing the infrastructure for patient safety education and culture-building. Risk management has had reactive and proactive approaches including adverse event reporting and learning, root cause investigation and failure mode and effect analysis.

### Values and commitments of the System

Patient safety enhancement, learning from events and errors, providing feedback to health care workers and confidentiality were four basic principles of the system that have been emphasized. Root cause analysis of reported events and other safety information and dissemination of results have met such important issues. Providing feedback has been done through safety alerts, presentation of notable cases in safety boards, and official informing of mentioned solutions to target groups. There were many problems in providing feedback. First, disseminating information in way that didn’t cause sham and blame and lead to disclosure of confidential data, needed to expert staff for providing reports, newsletters or alerts. Besides, high workload of most staff and large amount of documentation tasks have caused that patient safety alerts not to be listened. In order to overcome these problems, safety walkrounds with senior management was organized to emphasize on patient safety issues and its documents.

Priority of safety in hospital, make and maintain of nonpunitive approach, and provision of substantial resources and efficient staff, were the main hospital mandates. Patient safety information and reports was de-identified and no penal decisions were taken for reporters. Despite of system emphasizing on nonpunitive approach, some members of analysis teams and management staff have likewise focused on individual errors and necessity of organization encounter with mistakes in early stage of system implementation. With insistence of leadership on system based approach and search for system defects which underlie individual errors, gradually, this attitude has altered.

Fortunately, senior management of hospital through multiple meetings and discussion about safety data of hospital, had a good deal with system but there was some resistance in middle manager level that presented itself with lack of support of safety programs, resource limitation and punitive directions. Emphasizing on confidentiality and prioritizing of system failures, usage of national accreditation rules for safety requirements and reliance to leadership role of respective university were helpful for elimination of resistances.

### Patient safety education and culture-building

We put especial emphasis on different educational and training methods. Holding conferences, workshops, continuous and short training courses on different aspects of risk management system with the goal of educating all health care providers were the first step to define principles, concepts, and values of the system and culture-building promoting patient safety. Moreover, assisting the patient safety personnel to challengingly visit and auditing different health care delivery sectors and dialogue with the aim of learning from events and analyzing them was used as proactive education and culture building. Furthermore, safety walkrounds in the presence of senior managers of hospital in regular visits were designed and implemented. The main objective of this plan was demonstration of commitment of hospital management in terms of providing patient safety and provision of and monitoring its requirements. It is noteworthy that instructions to use different educational tools such as designing pamphlets and educational material were demonstrated. Despite of planning for education, participation of staff was weak and passive. It seems patient safety education should be consisted in work program of staff, or safety learning may be considered as a personal promotion indicator in future plans. High workload and organization culture, that safety doesn’t being prioritized, were the other reasons of weak participation. For evaluation of patient safety culture in mentioned hospital, a cross sectional survey was conducted using standard questionnaire of Hospital Survey on Patient Safety Culture from Agency for Healthcare Research and Quality that evaluates 12 patient safety culture dimensions and 2 outcomes. In total, 90 individual responded (overall response rate = 60 percent), including 64 nurses, 7 physicians, 19 of other staff.

### Reactive approach

In our experience, a voluntary adverse event reporting system was implemented in hospital. Reporting was performed mainly in two different ways of voluntary report using forms and secondly, documenting the issue and its consequences in patient safety log. Moreover, fatal and serious events should be reported to clinical governance and patient safety department by the head nurse to be followed up. After in receipt of the report and prioritizing of them, primary intervention measures were conducted in order to describe the detail of the incident thorough interviews with the responsible personnel, local inspection and reviewing records. Following data compiling, an expert assembly was formed to analyze the incident in depth and suggest strategies to overcome insufficiencies and defects; and ultimately, they supervised their implementation and outcome. Table 1 shows the aggregated data from all hospital wards. The participation of different wards in reporting process has extremely varied that might be produced by difference of clinical areas and workloads, faculty attitudes and cultural factors. In order to overcome low participation in some wards, several walkrounds with senior management of hospital were considered to promote patient safety and reporting culture. Since underreporting was predictable, internal alternative sources of safety information were also used for achieving event information. The mortality and morbidity board that inspect all expired patients, in previous 6 mounts, referred 33 suspicious cases to patient safety office for complementary investigation and root cause analysis. Besides, The committee of complains referred cases which correlated with medical errors and complains or self consent for discharge. Malpractice claims from external authorities were also started as a patient file and promptly conducted an investigation, interviewing all personnel involved to understand and correctly document exactly what happened.

**Table 1:** Reported adverse events in 18 months

** Type of adverse event**	**Emergency ward**	**Internal med. Wards**	**Surgery wards**	**Intensive care wards**	**Total**
Number of wrong infusion	3	21	9	37	70
Number of unsafe patient transport	4	10	1	6	21
Number of wrong sampling	14	5	2	17	38
Number of Unsuccessful urinary catheterization	8	9	0	4	21
Number of Unsuccessful IV- line catheterization	17	87	32	20	156
Number of urinary tract rupture during catheterization	1	3	0	0	4
Number of Bed sore occurred in hospital	131	503	245	66	945
Number of burning induced cautery	0	2	2	39	43
Number of adverse event from intubation	0	2	2	6	10
Number of falling	4	24	8	0	36
Number of kardex mistakes	113	0	0	31	144
Number of transfusion errors	1	1	0	1	3
Number of other reported events	0	190	33	228	451
Total	296	857	334	455	1942

Although reporting was voluntary, providing root cause analysis of catastrophic events which accompanied by an action plan was required. Expert group of RCA consisted of patient safety staff, clinical care provision teams, nursing staff, and managers of the hospital. Root cause analysis of most reported sentinel events was regularly conducted to identify human, organizational, and technical factors and to uncover the underlying systems failures, with the goal of redesigning system to reduce the likelihood of patient injury, and finally the results were fed back to caregivers and external authority in case of health care deputy of university.

Inadequate participation of staff in reporting and analyzing, because of fear of being blame and expectation that reports are ineffective, and limitation of resources and resistance of hospital management for solutions were the main executive problems. Also high turnover of medical staff, residents and interns, was considered as a reason of low participation of medical group in risk management processes. It seems that patient safety issues should be included in educational curriculum of medical students.

### Proactive approach

Using the technique of Failure Mode and Effect Analysis can be considered as a step-by-step approach to recognize different modes of the potential errors and failures in clinical service delivery. In this study, different interactions and functions of health care delivery in hospital are prioritized based on their importance and are systematically analyzed to define potential errors and malfunctions leading to the events, assess probable their consequences and distinguish contributory factors. Consequently, FMEA team, which was comprised of expert staff who were familiar with considered procedure, was committed for finding strategies to deal with predictable errors and control of their consequences. Therefore, this was highlighted for quality improvement as an ongoing process of risk management system. Organizing FMEA teams in each ward with faculty supervision was the final recommendation that could prevent coordination and orientation problems.

## Results

The findings of reporting activities in defined framework of risk management are shown in Table 1.

According to number of patients, the incidence rate of adverse events was calculated to 3.6% of hospitalized patients that is much less than expected rates.

A root cause analysis was conducted for some cases which referred from mortality board of hospital (33 cases). Although due to underreporting, the referred and reported events could not provide a representative database for patient safety accidents in hospital, however, this can be concluded that at least 5.3 percent of deaths in hospital related with adverse events. On the other hand, in at least 0.2 percent of hospitalized patients, adverse events played a role in patients death in past 6 mounts.

The results of survey on patient safety culture in hospital indicate that the patient safety culture scores were considerably low (average score of 12 dimensions of patient safety culture was 46.2 percent). The lowest scores were “non-punitive responses to error” (21.2 percent), “staff and related subjects” (26.1 percent), “teamwork across hospital units (29.1 percent) and “management support for patient safety” (29.7 percent). The dimension “teamwork within hospital units” generated the highest score (69.9 percent). In addition, 44.3 percent of the staff of hospital graded the safety performance of hospital as very good. Moreover, no events were reported by 57 percent of respondents during past 12 month (Table 2).

**Table 2:** Positive scores of patient safety culture dimensions

	
Teamwork within units	69.86
Supervisor/manager expectations & actions promoting patient safety	51.91
Organizational learning and continuous improvement	67.90
Management support for patient safety	29.69
Overall perceptions of patient safety	44.63
Feedback & communication about error	65.93
Communication openness	50.91
Frequency of events reported	50.33
Teamwork across units	29.09
Staffing	26.05
Handoffs & transitions	46.39
Nonpunitive response to errors	21.19

## Discussion

Health and clinical service delivery organizations are obliged to provide a safe environment for patients as well as staff [23]. Several different studies revealed that risk management is the basis for minimization of medical errors and enhancement of patient safety in hospitals which needs to be implemented as strategies and practical plans; and, simultaneously, clinical staff should be trained and well oriented of different risk management guidelines and scheme [24-26]. The results of a study in Iran indicated that no minimum risk reduction requirements are complied with in different wards of hospitals. Therefore, different risk assessment plans and also method for staff training and supervision was suggested [27]. Furthermore, Verbano et al., having assessed human errors and validity of risk management in health care provision institutes in Italy, concluded that attitudes and cultures towards risks and its management measures can differ vastly from one to another. Therefore, patient safety culture should be developed based on clinical governance policy and programs, comprehensive and short courses for risk management training, and implementation of clinical risk management [28]. Results of different research studies have demonstrated that educating staff regarding safety measures can lead to patient safety improvement [29]. The findings of another study indicated that a 4 weeks training program on safety significantly improved judgment and understanding of nurses, and as a result, they adhered more strictly to safety measures [30]. The leader and the manager of the organization or hospital play a key role in implementation of different safety measures through high priority of safety [31]. The results of risk management study in Baghiatollah hospital in 2007 demonstrated that patient safety improvement in hospitals requires a systematic approach and involvement of senior managers of hospital in safety management systems and their strict commitment [32]. In report of Surveillance Systems for Adverse Events and Medical Errors in the Unites States, there were many possible explanations for underreporting. The most commonly mentioned ones included: the fear of being blamed, the possibility of legal liability, and an expectation that reports will be futile. Moreover, the necessity of establishment of a confidential environment without “blame and shame” culture was highlighted [33]. Study on 700 hospital beds in 2007, revealed that achievement to an acceptable safety level in hospitals needs a close working relationship between clinical staff and support teams of hospitals [34]. In a different study in 44 hospitals of Pennsylvania in 2005; it was also concluded that for enhancement of patient safety, structural and organizational reforms and such as improvements in staff training programs, management information system and improvement of workplace situations is necessary, and this can only be successfully achieved when it is fully supported by the hospital management and well funded [35]. In all hospitals, a safety guideline needs to be designed and supported by high ranking officials and the ultimate goals and objectives need to be clearly defined [36].

A study on patient safety culture at the similar hospitals in Iran revealed that safety culture score in 10 dimensions is low to moderate, and lowest score was in nonpunitive response to errors and teamwork between hospital wards [37]. This subject was noticeable in our hospital which was presented in inappropriate time apportion and weak participation in reporting and analyzing patient safety information by all staff. This has been usually correlated with useless of data gathering.

Frankel and et al. indicate that safety walkrounds appears to be an effective tool for identifying safety issues, engaging leadership, and supporting a culture of safety [38]. His study has revealed that safety climate scale scores in hospitals have been increased 18 months post-walkrounds implementation. Walkround implementation requires significant organizational will [39]. Safety walkrounds helps educate leadership and frontline staff in patient safety issues and results in cultural changes, as manifested in more open discussion of adverse events and an improved rate of safety-based changes [40].

Weakness of patient safety culture and low knowledge resulted in weak participation in reporting and analysis. Uselessness of aggregated data and dominance of blame and penalty culture in hospital brought about under reporting and hiding events. Unremarkable commitment of hospital management on patient safety measures and monitoring of activities in this field led to ineffectiveness of safety improvement. Thus hospital risk management system should be focused on education and culture building in hospital. Furthermore, according to dominance of blame culture and existing tendency for hiding of information by all caregivers and also weakness of hospital information infrastructure, the voluntary reporting system was considered as the basis of data collection process. Because of predictable underreporting, other sources of information such as mortality and morbidity board, committee of complains and Malpractice claims were perceived. Because of cultural issues and expected loss of information, exploit of a proactive approach was considered necessary which applied by establishment of FMEA teams. However, the model introduced in this article can provide a practical framework in risk management system at the national level and for developing countries, especially at the initial steps of their system development.

## Competing interests

The authors declare that they have no competing interests.

## Authors’ contribution

HA: had the main role in design, implementation of this project, performance analysis of data and writing of manuscript. NK: supervisor of this project and deliver advantage guidance to progress this idea. HR: the corresponding author of this paper and had a benefit guidance in selecting methods and approved this idea. MJ and ARJ: had a good cooperation in this project and data gathering. All authors read and approved the final manuscript.

## References

[B1] Smits M Zegers M Groenewegen P Timmermans DRM Zwaan L van der Wal G Wagner C Exploring the causes of adverse events in hospitals and potential prevention strategies Qual Saf Health Care 2010 19 5 e5 10.1136/qshc.2008.030726 20142403

[B2] Zegers M Bruijne M Wagner C Groenewegen P Waaijman R van der Wal G Design of a retrospective patient record study on the occurrence of adverse events among patients in Dutch hospitals BMC Health Services Research 2007 25 7 27 10.1186/1472-6963-7-27 PMC1810530 17319971

[B3] Baker GR Norton PG Flintoft V Blais R Brown A Cox J Etchells E Ghali WA Hebert P Majumdar SR O’ Beirne M Palacios-Derflingher L Reid RJ Sheps S Tamblyn R The canadian Adverse Events Study: the incidence of adverse events among hospital patients in Canada CMAJ 2004 170 1678 1686 10.1503/cmaj.1040498 15159366 PMC408508

[B4] Brennan TA Leape LL Laird NM Hebert L Localio AR Lawthers AG Newhouse JP Weiler PC Hiatt HH Incidence of adverse events and negligence in hospitalized patients. Results of the Harvard Medical Practice Study I N Engl J Med 1991 324 370 376 10.1056/NEJM199102073240604 1987460

[B5] Davis P Lay-Yee R Briant R Ali W Scott A Schug S Adverse events in New Zealand public hospitals I: occurrence and impact N Z Med J 2002 115 U271 12552260

[B6] Hayward RA Hofer TP Estimating hospital deaths due to medical errors: preventability is in the eye of the reviewer JAMA 2001 286 415 420 10.1001/jama.286.4.415 11466119

[B7] Jarman B Gault S Alves B Hider A Dolan S Cook A Hurwitz B Iezzoni LI Explaining differences in English hospital death rates using routinely collected data BMJ 1999 318 1515 1520 10.1136/bmj.318.7197.1515 10356004 PMC27892

[B8] Michel P Quenon JL de Sarasqueta AM Scemama O Comparison of three methods for estimating rates of adverse events and rates of preventable adverse events in acute care hospitals BMJ 2004 328 7433 199 10.1136/bmj.328.7433.199 14739187 PMC318484

[B9] Park RE Brook RH Kosecoff J Keesey J Rubenstein L Keeler E Kahn KL Rogers WH Chassin MR Explaining variations in hospital death rates. Randomness, severity of illness, quality of care JAMA 1990 264 484 490 10.1001/jama.1990.03450040080035 2195173

[B10] Schioler T Lipczak H Pedersen BL Mogensen TS Bech KB Stockmarr A Svenning AR Frolich A Danish Adverse Event Study: [Incidence of adverse events in hospitals. A retrospective study of medical records] Ugeskr Laeger 2001 163 5370 5378 11590953

[B11] Thomas EJ Studdert DM Burstin HR Orav EJ Zeena T Williams EJ Howard KM Weiler PC Brennan TA Incidence and types of adverse events and negligent care in Utah and Colorado Med Care 2000 38 261 271 10.1097/00005650-200003000-00003 10718351

[B12] Vincent C Neale G Woloshynowych M Adverse events in British hospitals: preliminary retrospective record review BMJ 2001 322 517 519 10.1136/bmj.322.7285.517 11230064 PMC26554

[B13] Wilson RM Runciman WB Gibberd RW Harrison BT Newby L Hamilton JD The Quality in Australian Health Care Study Med J Aust 1995 163 458 471 7476634 10.5694/j.1326-5377.1995.tb124691.x

[B14] Vries E Ramrattan MA Smorenburg SM Gouma DJ Boermeester MA The incidence and nature of in-hospital adverse events: a systematic review. Qual Saf Health Care 2008 17 216 223 10.1136/qshc.2007.023622 18519629 PMC2569153

[B15] Hoonhout LH de Bruijne MC Wagner C Direct medical costs of adverse events in Dutch hospitals BMC Health Services Research 2009 9 27 10.1186/1472-6963-9-27 19203365 PMC2645386

[B16] Zegers M de Bruijne MC Wagner C Adverse events and potentially preventable deaths in Dutch hospitals: results of a retrospective patient record review study Qual Saf Health Care 2009 18 297 302 10.1136/qshc.2007.025924 19651935

[B17] Zegers HWM Wagner C Adverse events among hospitalized patients: results and methodological aspects of a record review study Program coordinator 2009 NIVEL: PhD thesis Utrecht 207

[B18] World Health Organization World alliance for patient safety WHO Draft Guidelines for Adverse Event Reporting and Learning Systems From information to action 2005 Geneva http://www.who.int/patientsafety/events/05/Reporting_Guidelines.pdf

[B19] Kohn LT To err is human: building a safer health care system 1999 Institute of Medicine 1 11 http://books.nap.edu/openbook.php?isbn=0309068371

[B20] World Health Organization World Alliance for Patient Safety Forward Programme 2005 Geneva http://www.who.int/patientsafety/en/brochure_final.pdf

[B21] The Joint Commission Improving America’s Hospitals The Joint Commission’s Annual Report on Quality and Safety 2007 http://www.jointcommission.org/assets/1/6/2007_Annual_Report.pdf

[B22] Tasmanian CT review team Risk management process Draft guidance manual for infrastructure operators 2003 http://www.statesecurity.tas.gov.au/docs/riskmanagement.pdf

[B23] Hare C Davies C Shepherd M Safer medicine administration through the use of e-learning Nursing Times 2006 102 16 25 27 16669205

[B24] Neale G Risk management in the care of medical emergencies after referral to hospital J R Coll Physicians Lond 1998 32 2 125 9 9597627 PMC9663020

[B25] Neale G Woloshynowych M Vincent C Exploring the causes of adverse events in NHS hospital practice J R Soc Med 2001 94 322 30 11418700 10.1177/014107680109400702 PMC1281594

[B26] Handel DA McConnell KJ Emergency department length of stay and predictive demographic characteristics Ann Emerg Med 2007 50 3 s70

[B27] Yarahmadi R Performance assessment on hospital inpatients departments based on rules and policy Health Work J 2009 3 6 1 5

[B28] Verbano C Turra F A human factors and reliability approach to clinical risk management: Evidence from Italian cases Saf Sci 2010 48 5 625 39 10.1016/j.ssci.2010.01.014

[B29] Colorado Center for Nursing Excellence The 2004 Colorado. Nursing Faculty Supply and Demand Study 2007 http://www.coloradonursingcenter.org

[B30] National League for Nursing Challenges to the Nursing Discipline 2003 2003 http://www.nln.org

[B31] Moi Lin L Healthcare Worker Safety and Welfare: Responsibility of the Hospital Administration Int J Antimicrob Agents 2005 26 1 S1 S6 10.1016/j.ijantimicag.2005.04.013 16291480

[B32] Zaboli R Tofighi S Delavari A Mirhashemi S Survey of Safety Management On Bagiyatallah (a.s) Hospital, 2006–07 J Mil Med 2007 9 2 103 111

[B33] United States General Accounting Office ADVERSE EVENTS, Surveillance Systems for Adverse Events and Medical Errors 2000 http://www.gao.gov/assets/110/108220.pdf

[B34] Patton JM Hoffmann KT Patient Precautions Task Force-Hand Off Communication for Patient Safety in a 700-Bed Community Hospital System Am J Infect Control 2007 35 5 E137

[B35] Anderson JG Ramanujam R Hensel D Anderson MM Sirio CA The need for organizational change in patient safety initiatives Int J Med Inform 2006 75 12 809 817 10.1016/j.ijmedinf.2006.05.043 16870501

[B36] Wood RH Aviation Safety Programs: A Management Handbook 2003 Jeppesen Sanderson: Third edition

[B37] Abdi Z Maleki MR Khosravi A Perceptions of patient safety culture among staff of selected hospitals affiliated to Tehran University of Medical Sciences Payesh 2011 10 4 411 19

[B38] Frankel A Grillo SP Baker EG Huber CN Abookire S Grenham M Console P O'Quinn M Thibault G Gandhi TK Patient Safety Leadership WalkRounds at Partners Healthcare: learning from implementation Jt Comm J Qual Patient Saf 2005 31 8 423 37 16156190 10.1016/s1553-7250(05)31056-7

[B39] Frankel A Grillo SP Pittman M Thomas EJ Horowitz L Page M Sexton B Revealing and resolving patient safety defects: the impact of leadership WalkRounds on frontline caregiver assessments of patient safety Health Serv Res 2008 43 6 2050 66 10.1111/j.1475-6773.2008.00878.x 18671751 PMC2613998

[B40] Frankel A Graydon-Baker E Neppl C Simmonds T Gustafson M Gandhi TK Patient Safety Leadership WalkRounds Jt Comm J Qual Saf 2003 29 1 16 26 12528570 10.1016/s1549-3741(03)29003-1

